# An improved sequencing-based strategy to estimate locus-specific DNA methylation

**DOI:** 10.1186/s12885-015-1646-6

**Published:** 2015-09-21

**Authors:** Giulia Brisotto, Alessandra di Gennaro, Valentina Damiano, Michela Armellin, Tiziana Perin, Roberta Maestro, Manuela Santarosa

**Affiliations:** 1 Experimental Oncology 1, CRO Aviano National Cancer Institute, via F. Gallini 2, Aviano, 33081 PN Italy; 2 Pathology, CRO Aviano National Cancer Institute, via F. Gallini 2, Aviano, 33081 PN Italy

**Keywords:** DNA-methylation, miR-200c/miR-141 locus, Method, Cancer, Bisulfite treatment, Sequencing, E-cadherin, CDH1, Promoter

## Abstract

**Background:**

DNA methylation is an important epigenetic mechanism of transcriptional control that plays an essential role in several cellular functions. Aberrant DNA methylation in cancer has been frequently associated with downregulation of microRNAs and protein coding genes, such as miR-200c/miR-141 cluster and E-cadherin. Current strategies to assess DNA methylation, including bisulfite treatment-based assays, tend to be time-consuming and may be quite expensive when a precise appraisal is required. The Sanger-sequencing of the amplified bisulfite-treated DNA (BSP) might represent a practical option to measure DNA methylation at single CpG resolution. However, this strategy often produces noisy data, which affects accurate quantification. Here we propose an improved, reliable and cost-effective BSP-based protocol that allows proper DNA methylation assessment.

**Methods:**

Our strategy, named normalized-BSP (NBSP), takes advantage of tailed C-balanced primers and a normalization procedure based on C/T ratio to overcome BSP-associated noise problems and nucleotide signal unbalance. NBSP was applied to estimate miR-200c/miR-141 locus methylation in serial dilution experiments and was compared to conventional methods. Besides, it was applied in the analysis of FFPE breast cancer samples and further validated in the context of the E-cadherin promoter.

**Results:**

NBSP strategy outperformed conventional BSP in the estimate of the fraction of methylated cytosine in serial dilution experiments, providing data in agreement with the widely used but cumbersome cloning-based protocol. This held true for both miR-200c/miR-141 locus and E-cadherin promoter analyses. Moreover, the miR-200c/miR-141 locus methylation reflected the decrease in miRNA expression both in breast cancer cell lines and in the FFPE samples.

**Conclusions:**

NBSP is a rapid and economical method to estimate the extent of methylation at each CpG of a given locus. Notably, NBSP works efficiently on FFPE samples, thus disclosing the perspective of its application also in the diagnostic setting.

**Electronic supplementary material:**

The online version of this article (doi:10.1186/s12885-015-1646-6) contains supplementary material, which is available to authorized users.

## Background

DNA methylation, one of the best-characterized epigenetic modifications, consists in the addition of a methyl group to cytosines included in CpG dinucleotides. The methylation of CpG islands (CGI), which are common in promoter regions, correlates with gene transcriptional repression [1, 2]. Aberrant DNA methylation is typically observed in tumors where it occurs at both protein coding gene and microRNA (miRNA) loci [3–5].

Several technologies have been developed to profile the methylation at CGI. These include comprehensive but expensive next-generation sequencing-based approaches (i.e.: WGBS [6, 7], RRBS [8], MethylCap-seq [9] or MBD-seq [10] as well as array- and PCR-based methods, more affordable and still used [11, 12]. Most techniques rely on the bisulfite conversion of unmethylated cytosine to uracil, and thus to thymine after PCR, leaving unaltered the methylated cytosine [13].

Rapid and simple methods to detect the ratio between C and T include the Sanger sequencing of PCR products of bisulfite-treated DNA (BSP). However, this approach fails to provide a quantitative measure of methylation because of high background noise and overscaled cytosine signals due to the DNA sequencing software that artificially adjusts signal strengths of underrepresented bases [14]. On the other hand, the cloning and subsequent Sanger sequencing of the PCR clones (cloning-based sequencing method) [15], although more accurate, is time-consuming and expensive, as it needs the sequencing of a significant number of clones for statistical accuracy [16].

Here we report an enhanced Sanger sequencing-based protocol for quantifying CGI promoter methylation based on the use of 5’-end tailed PCR primers that allow for the improvement of both signal-to-noise and C/T ratio. The method was successfully applied to assess the methylation status of both a miRNA locus (miR-200c/miR-141) and the promoter of E-cadherin and was also suitable for the analysis of FFPE tumor samples.

MiR-200 is a tumor suppressor miRNA family that includes five members clustered and expressed as two separate polycistronic pri-miRNAs: the miR-200a/miR-200b/miR-429 cluster, mapping at 1p36; and the miR-200c/miR-141 cluster, at 12p13 [17–19]. Promoter hypermethylation has been reported to play a crucial role in the downregulation of miR-200 [20–22] that has been associated with malignancy, increased chemo- and radio-resistance, invasiveness and transition of carcinomas from epithelial towards a mesenchymal phenotype (EMT) [23–29]. A hallmark of EMT is the downregulation of the cell-cell adhesion protein E-cadherin (E-cad) [30], whose low expression, as a result of promoter hypermethylation, has been described in diverse carcinoma subtypes and is associated with poor prognosis [31–33].

## Methods

### Cell lines

MDA-MB-231, MDA-MB-157 and MCF7 were obtained from ATCC (American Type Culture Collection) and cultured as previously described [34].

### Patients and samples

Formalin-fixed paraffin-embedded (FFPE) specimens from 3 breast cancers were collected at the CRO Aviano National Cancer Institute Biobank under patients’ informed consent. The use of tumor samples for this study was approved by the Institutional Review Board. Two 20 μm-slides with tumor cellularity greater than 70 %, as evaluated by a breast cancer pathologist (TP), were used per each case. Total RNA and DNA were isolated using the Recover All Total Nucleic Acid Isolation Kit (Life Technologies) according to the manufacturer’s instructions.

### RNA extraction and qRT-PCR

Total RNA from cell lines was isolated using TRIzol (Life Technologies). MiRNA was reverse-transcribed and qRT-PCR performed using the TaqMan MicroRNA Assay kits specific for miR-200c and RNU48 (Life Technologies) and TaqMan Universal Master Mix (Life Technologies) according to the manufacturer's guidelines. miRNA levels were normalized with RNU48 and relative levels were calculated using the ΔΔCt method. Three independent experiments were performed in triplicate.

### DNA extraction and bisulfite conversion

Genomic DNA was extracted from cell lines using the EZ1 DNA Tissue Kit (Qiagen). Bisulfite conversion of DNA (500 ng - 1 μg), obtained from cell lines and tissues, was carried out with the EpiTect Bisulfite kit (Qiagen), according to the manufacturer’s instructions.

### Bisulfite PCR amplification

**Fig. 1:**
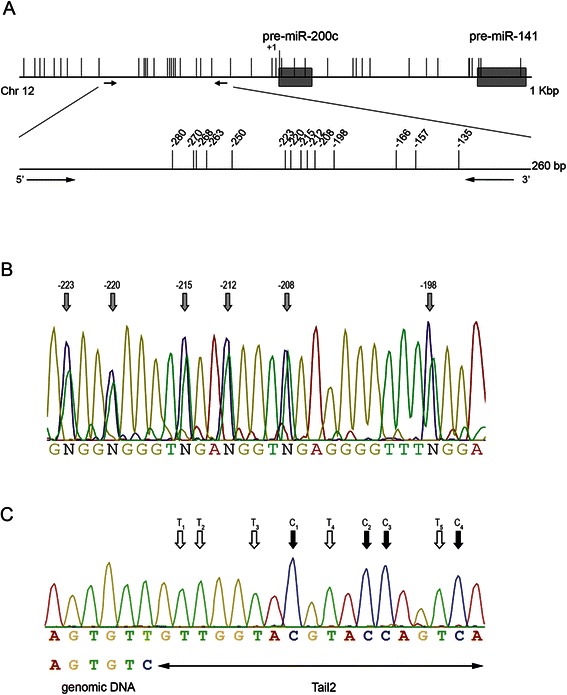
Schematic diagram of the miR-200c/miR-141 locus and representative chromatograms of the PCR products. **a** Graphical depiction of the miR-200c/miR-141 genomic locus showing individual CpG sites as vertical lines and the pre-miR-200c and pre-miR-141 sequences as gray boxes. Arrows indicate the location of primers and delimitate the analyzed CGI that encompasses the region from −353 to −108 nucleotides, relative to the first nucleotide of the pre-miR-200c. The bottom bar is an enlargement of the analyzed CGI. **b** Representative sequencing chromatogram of the amplicon obtained from 25 % plasmid standard by using untailed primers (200c-BSP-F and 200c-BSP-R). Six out of 14 CpG are reported and indicated by *gray arrows*. **c** Part of the sequencing chromatogram of the Tail1-200c-F/Tail2-200c-R amplicon showing the Tail2 region. The *black arrows* indicate the C and the *white arrows* the T whose peak heights were used to determine the NF

The region of the miR-200c/miR-141 locus, spanning from position −353 to −108 relative to the pre-miRNA-200c first nucleotide (chromosome12:7,072,510:7,072,755; Fig. 1a) and the promoter region from −115 to +54 nucleotide relative to the transcriptional start site of E-cad (CDH1 gene; chromosome16:68,771,079: 68,771,249; Fig. 5a) were amplified with primers specifically designed by MethPrimer (Additional file 1) [35].

5’-end tailed primers were obtained by adding at the 5’-end of the 200c-BSP-F and 200c-BSP-R a tail derived from the M13 (Tail1) or from the Decipher Project barcode library (Tail2-6; http://www.decipherproject.net). Tail1, Tail3 and Tail5 were added to the forward oligo and Tail2, Tail4 and Tail6 to the reverse oligo (Additional file 1). Tails 2–6 were randomly chosen among barcodes devoid of C or G at the 5’-end and in which each base is roughly equally represented (22-28 %). The tails, by contributing with C and T (G and A in the reverse primer) allow for compensation in the elaboration process. Primers with Tail1, Tail3 and Tail5 were used in combination with primers with Tail2, Tail4 and Tail6, respectively. All the three couples of primers well amplified miR-200c/miR-141 locus (Additional file 2). We selected Tail1- and Tail2-primers for this work. Tail1 and Tail2 were also added to E-cad-BSP forward and reverse oligo, respectively.

0.7-1 μl of bisulfite-treated DNA were amplified by using GoTaq® Polymerase (Promega) if not otherwise specified. The PCR amplification was performed in 20 μl reaction volume containing GoTaq® Green Master Mix 1X, 250 nM forward and reverse primers and with the following protocol: 95 °C for 4 min, 40 X [95 °C for 45 s, 60 °C (E-cad) or 62 °C (miR-200c) for 1 min and 30 s, 72 °C for 1 min 30 s], 72 °C for 4 min. Phusion U Hot Start DNA polymerase (Thermoscientific) was tested for the amplification of miR-200c/miR-141 locus of genomic DNA standards. The PCR amplification of 20 μl mixture containing 0.7-1 μl of bisulfite-treated DNA, 0.4 U Phusion U Hot Start DNA Polymerase, 400 nM forward and reverse primers, Phusion HF Buffer 1X and 200 μM dNTPs was performed with the following protocol: 98 °C for 1 min, 37 X [98 °C for 10 s, 64 °C for 15 s, 72 °C for 30 s] 72 °C for 5 min. 10 μl of PCR products were size-checked on a 2 % agarose gel and 5 μl were purified with 2 μl of ExoSap-IT (Affymetrix).

### PCR cloning and assessment of methylation

Bisulfite-treated DNA was amplified by PCR with untailed primers (Additional file 1) and 1 μl of the PCR was directly cloned into the pCR2.1 vector using TA Cloning Kit (Life Technologies), according to the manufacturer’s protocol. Plasmids DNA from at least 20 colonies were isolated using the QiaPrep Spin Plasmid Miniprep kit (Qiagen) and sequenced. The methylation level for each CpG was deducted by dividing the number of C at each CpG site for the total number of clones sequenced.

### Generation of DNA standards

We generated plasmid and genomic DNA standards to mimic different methylation levels of miR-200c/miR-141 locus. To obtain the plasmid DNA standards, miR-200c/miR-141 locus was amplified from bisulfite-converted DNA of MCF7 and MDA-MB-157 (unmethylated and 97 % methylated, respectively, as determined by the cloning method) and cloned into the pCR2.1 vector (TA Cloning Kit, Life Technologies) according to the manufacturer’s protocol. Two of these clones derived from completely methylated and unmethylated (for all CpG sites) template, respectively, were mixed to mimic different DNA methylation percentages: 0, 12.5, 25, 55, 75, 87.5 and 100 %. The C/T ratio, calculated as described below, was confirmed by plasmid direct sequencing (Additional file 3).

Moreover, a set of the genomic DNA standards was generated by mixing the bisulfite-treated DNAs of the aforementioned cell lines in order to obtain the following methylation levels: 0, 12.1, 24.2, 48.4, 72.6 and 97 %.

### Sequencing

Sequencing reactions (10 μl) were performed using 1 μl of ExoSap-IT-purified PCR amplicons or 500 ng of plasmids, 2 μl of BigDye Terminator v.3.1 kit (Life Technologies), 300 nM sequencing primer, corresponding to 200c-BSP-F, Tail1 or Tail2 (Additional file 1), and the following protocol: 95 °C for 5 min, 25 X [95 °C 30 s, 50 °C for 30 s and 60 °C for 1 min and 30 s]. The sequencing reactions were then purified using the BigDye XTerminator Purification kit and ran on an ABI prism 3130 Genetic Analyzer (Applied Biosystems). SeqScape® Software v2.5 with the KB™ basecaller software or Chromas Lite Version 2.1.1 were used for data analysis.

### Quantification of methylation by BSP

DNA standards and bisulfite-treated DNA were amplified by PCR with tailed primers (Additional file 1) and sequenced as described. The percentage of methylation at each CpG site was calculated as 100 ∗ *C*/(*C* + *T*), i.e. 100 times the ratio between the peak height of C on the sequencing chromatograms and the sum of peak height for C and T [36].

### Quantification of methylation by NBSP

DNA standards and bisulfite-treated DNA were amplified by PCR with 5’-end tailed primers (Additional file 1) and sequenced as above. To adjust the overscaled C signals in the sequencing chromatograms we introduced a normalization factor (NF), based on the ratio of the signals for the C and T encoded by the tails of primers. Specifically, NF was calculated as the ratio between the mean of the peak height of the C and T read in sense direction on the sequence of Tail2 (corresponding to G and A in Tail2 reverse primer sequence; Additional file 1, Figs. 1c and 5c).

Then, the peak height of each C (C_i_) included in the target sequence was corrected for this NF as follow: C_norm_ = C_i_/NF. Finally, the normalized C signals were used to determine the methylation percentage as described above, i.e. 100 ∗ C_norm_/(C_norm_ + T).

### Statistical analyses

The concordance between observed and expected values was analyzed by using the approach recommended by Bland and Altman [37, 38]. For all Bland–Altman plots, the mean percentage difference between the observed and expected results (mean bias) with associated 97.5 % confidence intervals and limits of agreement (±1.96 SD) were calculated (GraphPad Prism software).

## Results

For the analyses of miR-200c/miR-141 promoter methylation we focused on the region referred to as relevant for transcription (−353 to −108, relative to the pre-miRNA-200c first nucleotide) and that comprised 14 CpG sites (Fig. 1a) [17, 18].

We first performed the analysis of this region in a set of plasmid DNA standards obtained by mixing defined amount of clones corresponding to methylated and unmethylated DNA (see Methods). The direct sequencing of the PCR products of these standards displayed overscaled C signals and a high background noise that prevented the actual estimate of miR-200c/miR-141 promoter methylation (Fig. 1b and Additional file 4A).

**Fig. 2:**
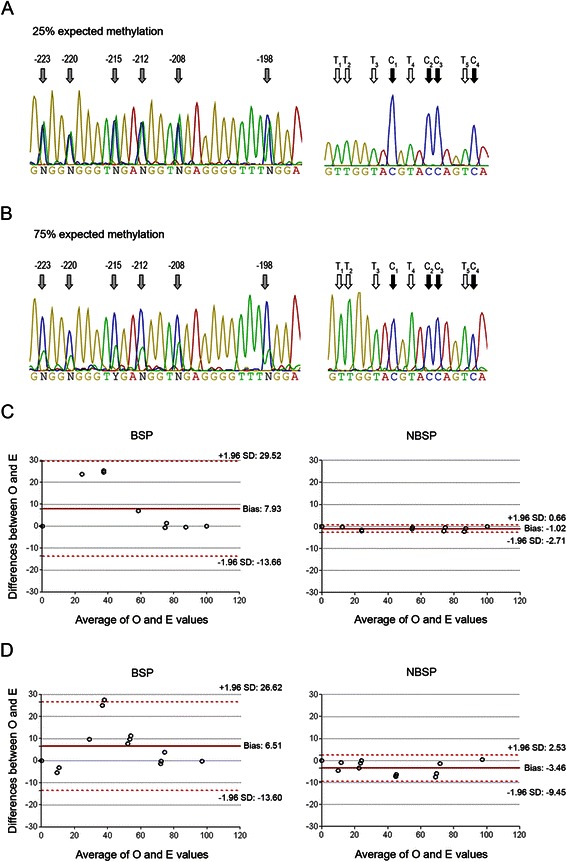
BSP and NBSP of plasmid and genomic DNA standards. Representative sequencing chromatograms of plasmid DNA standards characterized by 25 % (**a**) and 75 % (**b**) CGI methylation (see Methods). Each mixture was PCR amplified with the 5’-end tailed primers for miR-200c/miR-141 locus and the amplicons were sequenced using the Tail1 as a sequencing primer. *Left panels* depict 6 out of 14 CpG analyzed (indicated by *gray arrows*), while the *right panels* show the chromatograms relative to the Tail2-200c-R primer for miR-200c/miR-141 locus. C and T used to calculate the NF in the NBSP are highlighted by *black* and *white* arrows, respectively. **c**-**d** Bland–Altman plots of plasmid DNA standards (c) and genomic DNA standards (d) show the extent to which observed and expected methylation values of DNA standards agree. Methylation was evaluated by BSP (**c** and **d**, *left panels*) or NBSP (**c** and **d**, *right panels*). The solid lines represent the mean percentage difference between observed and expected (Bias) and the dashed lines ±1.96 SD of the mean percentage difference (limits of agreement). Filled circles represent individual measurements

In order to improve the quality of the sequencing traces, we amplified the aforementioned standards with 5’-end tailed primers (Fig. 1c and Additional files 1 and 4B) characterized by at least 4 C in the tails. The sequencing of these PCR products (BSP) provided chromatograms without any or only minimal background (Fig. 2a and b). Still, the measure of methylation extent was unsatisfactory. In fact, especially in the presence of low-intermediate levels of methylation, the C signals (i.e. non-converted, methylated cytosines) were overscaled, which resulted in an overestimate of DNA methylation. In fact, the mean bias (i.e. average percent difference between the observed and expected methylation levels) was 7.93 (limits of agreement from −13.66 to 29.52; Fig. 2c *left panel*). It is worth to note that the clone from unmethylated DNA displayed a G > A transition (at position 7,072,604 in the miR-200c/miR-141 locus). The ratio between G and A of each standard reflected the expected methylation levels suggesting the goodness of the standards (data not shown).

To overcome the C overestimation, we introduced a normalization strategy (referred in text as Normalized BSP, NBSP) that took into account the elaboration of overall nucleotide signals by the DNA sequencing software. Based on the assumption that, for any given sequence and in the absence of altering factors, the relation between mean of the peak height of two nucleotides, namely C and T, should be relatively constant, we calculated the ratio between C and T within the tail of the primers and used this ratio to normalize the overscaled C signals of the sequence (see Methods). The introduction of this normalization step significantly improved the estimate of methylation rate reducing the mean bias to −1.02 (Fig. 2c right panel; limits of agreement from −2.71 to 0.66).

Next we validated our strategy on the genomic DNA standards. Uracil present in the bisulfite-converted DNA may impair the DNA polymerase activity of Taq polymerase. Thus we compared the results obtained with Taq polymerase and with an uracil tolerant enzyme (Phusion U Hot start DNA polymerase). The two DNA polymerases showed similar results (Additional file 4C-F) and, importantly, NBSP displayed an improvement in the assessment of the methylation rate of genomic DNA standards compared to BSP in both analyses (Fig. 2d, Additional file 4G-H).

**Fig. 3:**
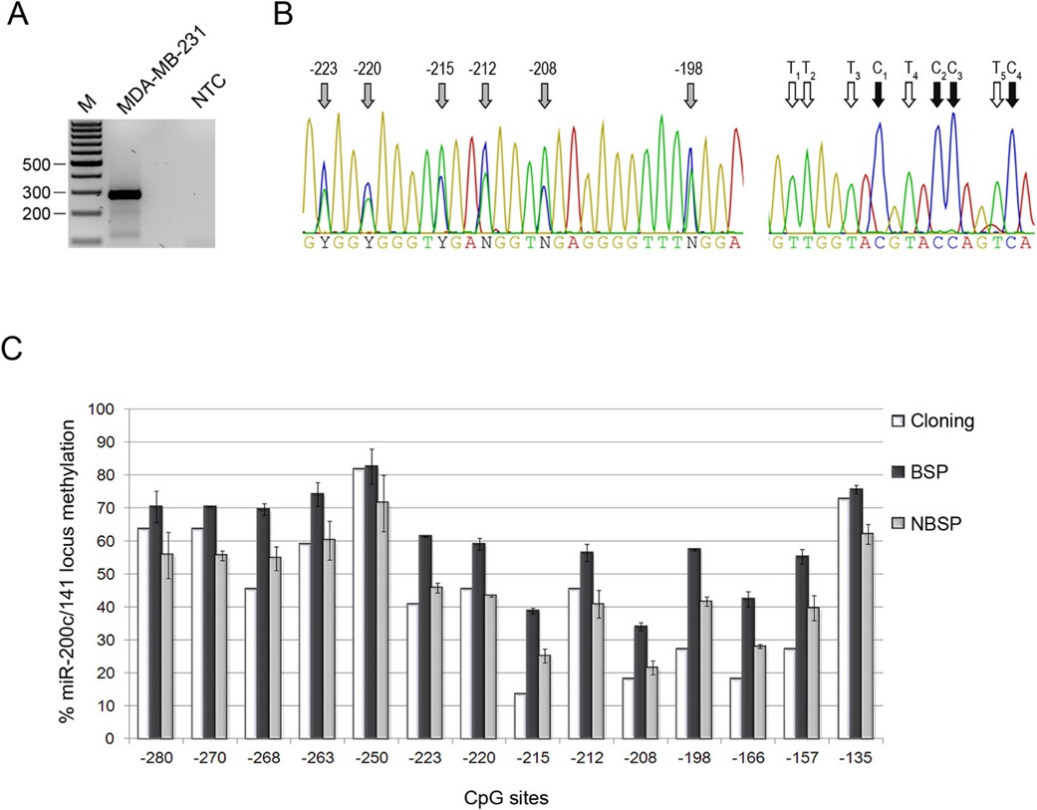
Comparison between BSP, NBSP and cloning-based methods in the analysis of miR-200c/miR-141 locus of MDA-MB-231 breast cancer cell line. **a** miR-200c/miR-141 locus PCR of bisulfite treated DNA obtained from MDA-MB-231. Lane M, 100 bp size marker. NTC, no template control. **b** Representative sequencing chromatogram of 6 CpG (highlighted by *gray arrows*; *left panel*) and of the reverse sequence of Tail2 of miR-200c/miR-141 amplicon (*right panel*). C and T used to calculate the NF are highlighted by *black* and *white arrows*, respectively. **c** The methylation percentages of each CpG obtained from the cloning-based method (22 clones sequenced, *white columns*), BSP (*black columns*) and NBSP (*gray columns*) are reported. BSP and NBSP were performed on three MDA-MB-231 samples. Bars correspond to standard deviation

To further validate our signal normalization approach, we compared the performance of BSP and NBSP to the cloning-based sequencing method. According to the standard BSP procedure, the percentages of methylation at each CpG of the miR-200c/miR-141 locus in the MDA-MB-231 breast cancer cell line ranged from ~ 30 to 80 %. These were globally greater than those gauged by the cloning-based method, particularly for low and intermediate CpG methylation (Fig. 3a-c). NBSP outperformed the BSP, providing estimate close to those of the cloning procedure for the majority of CpG sites. Forward and reverse tailed primers provided similar results, both in terms of percentages of methylation and extent of the normalization factors (Additional file 5).

The partial methylation of the miR-200c/miR-141 locus in MDA-MB-231 corresponded to a limited expression of miR-200c compared to the unmethylated MCF7 and the fully methylated MDA-MB-157 (Additional file 6).

**Fig. 4:**
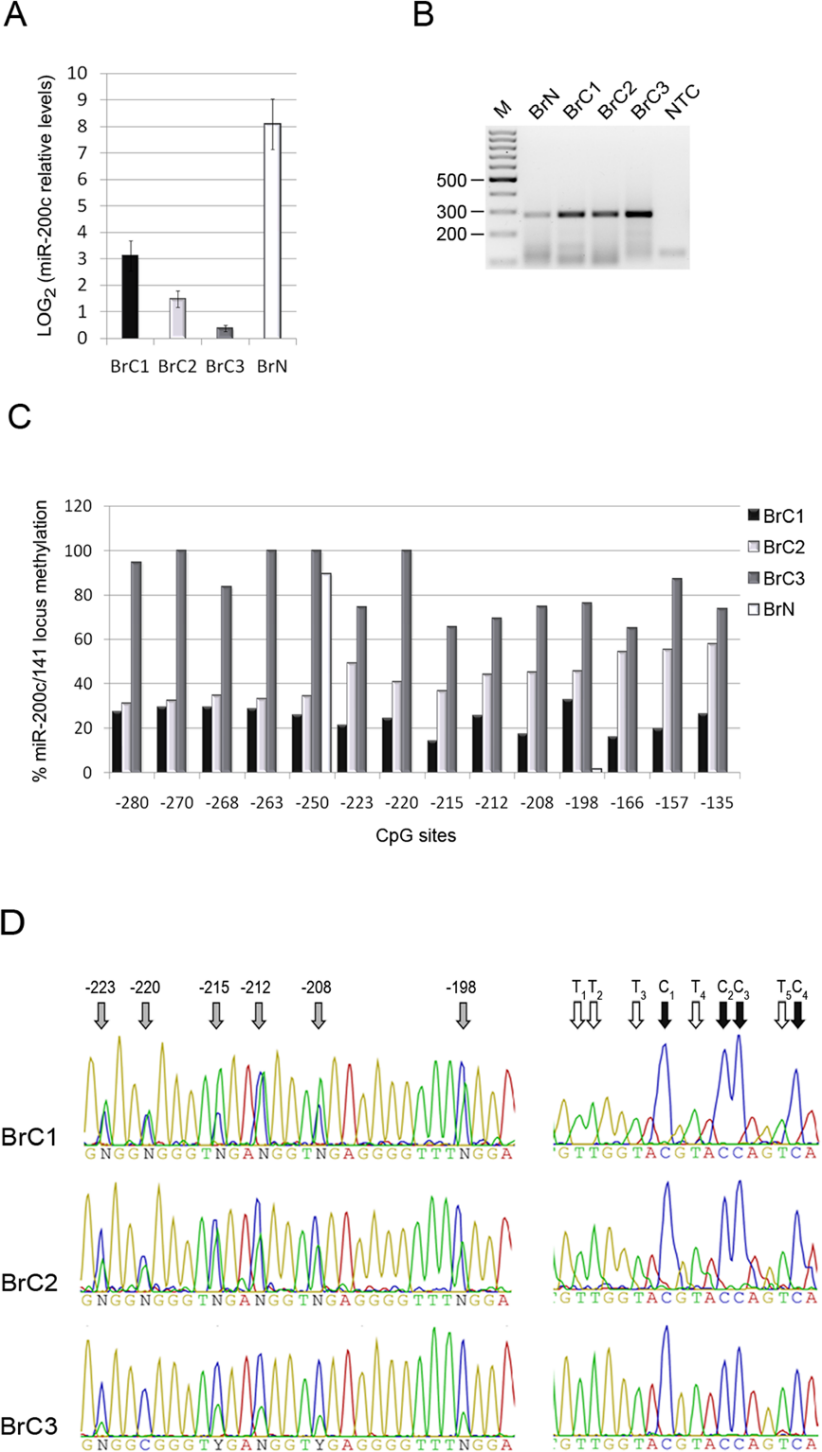
miR-200c expression and locus methylation of breast cancer tissues. **a** miR-200c expression levels of 3 FFPE breast cancers (BrC1, BrC2 and BrC3) and one FFPE normal tissue (BrN) were determined by qRT-PCR and reported as the LOG_2_ of the miR-200c levels relative to the RNU48 normalizer control. Data represent the means of three independent experiments performed in triplicate and bars indicate standard deviation. **b** miR-200c/miR-141 locus PCR of bisulfite treated DNA obtained from the FFPE breast normal tissue (BrN) and cancers (BrC1, BrC2, BrC3). Lane M, 100 bp size marker. NTC, no template control. **c** Methylation percentages of each CpG in the miR-200c/miR-141 locus of BrC1, BrC2, BrC3 and BrN. Data were obtained with NBSP. **d** Representative sequencing chromatograms of 6 CpG (highlighted by *gray arrows*; *left panels*) and of the reverse sequence of Tail2 of miR-200c/miR-141 amplicon (*right panels*; with C and T used to calculate the NF indicated by *black* and *white arrows*, respectively) for the three breast cancer samples

A similar inverse association between miR-200c expression and locus methylation was observed also when NBSP was applied to FFPE breast tumor samples, particularly for the CpG from −223 to −135 (Fig. 4a-d). A normal breast tissue sample showed only one methylated CpG and, as expected, expressed high levels of miR-200c.

**Fig. 5:**
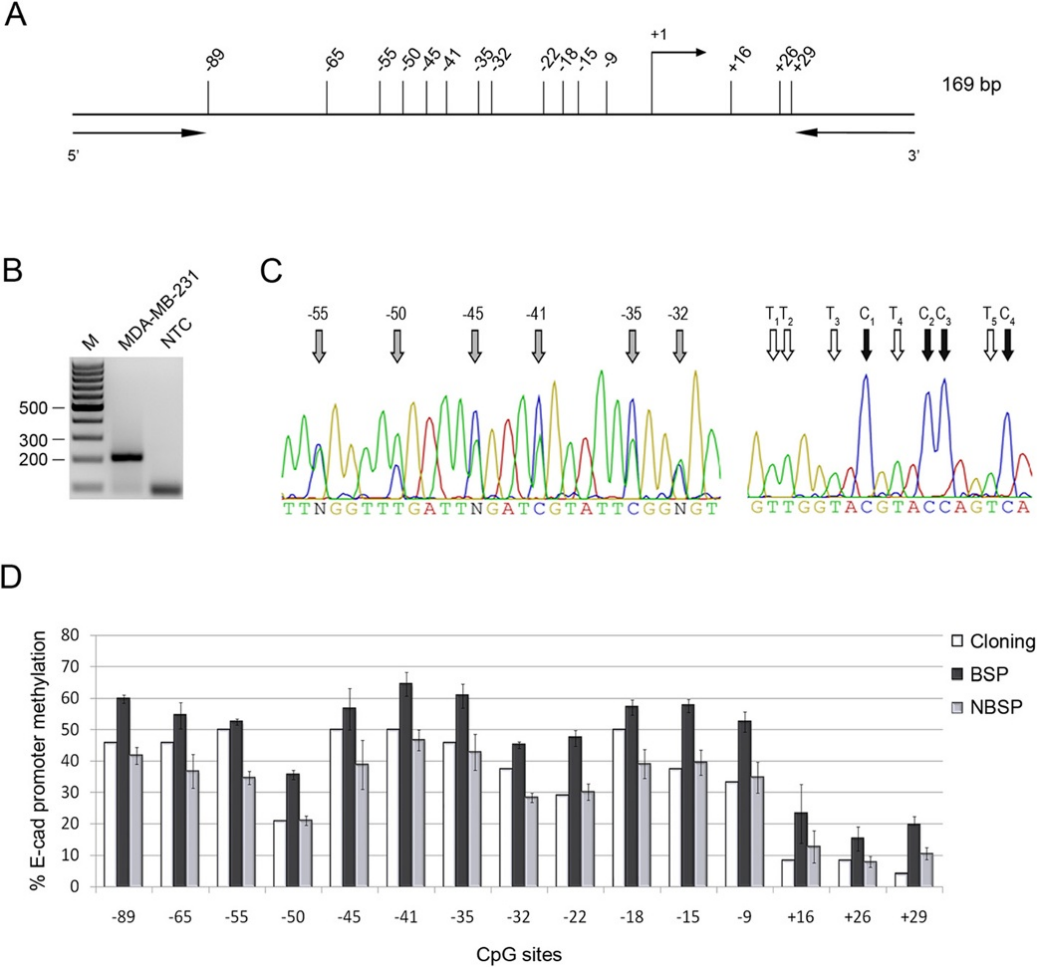
CGI methylation of E-cad promoter. **a** Schematic representation of the region within E-cad promoter spanning from −115 to +54 nucleotides, relative to the transcription start site (+1). Vertical lines represent each individual CpG and arrows indicate the location of primers. **b** E-cadherin promoter PCR of bisulfite treated DNA obtained from MDA-MB-231. Lane M, 100 bp size marker. NTC, no template control. **c** Representative sequencing chromatogram of 6 CpG (highlighted by *gray arrows*; *left panel*) and of the reverse sequence of Tail2 of the Tail1-Ecad-F/Tail2-E-Cad-R amplicon (*right panel*). C and T used to calculate the NF are highlighted by *black* and *white* arrows, respectively. **d** The graph reports the methylation percentages of each CpG (from −89 to +29) obtained from the cloning-based method (22 clones sequenced, *white columns*), BSP (*black columns*) and NBSP (*gray columns*). BSP and NBSP were performed on three MDA-MB-231 samples. Bars correspond to standard deviation

Finally, we investigated the methylation pattern of E-cad, a typical gene silenced by DNA-hypermethylation. Our study focused on a well-defined CGI spanning between −115 and +54 nucleotides from transcription start site of the E-cad promoter (Fig. 5a). Again, NBSP outperformed BSP in the measure of E-cad promoter methylation in MDA-MB-231 and provided data similar to those obtained with standard cloning-based method (Fig. 5b-d).

## Discussion

Epigenetic inactivation of tumor suppressor genes is a frequent event that drives tumorigenic initiation and progression [39–41]. The increasing interest in the evaluation of miR-200c/miR-141 locus methylation as a measure of cancer progression [42, 43], prompted us to set up a reliable, fast and affordable method for the assessment of DNA methylation.

The NBSP method here proposed relies on the use of 5’-end tailed primers that reintroduce ‘true’ C, improve the quality of sequencing traces and allow C/T signal normalization. We implemented the normalization procedure because of the overscaled C signals engendered by the sequencing software which, during raw data elaboration, tends to artificially enhance the signal of underrepresented C resulted from the bisulfite treatment. Overestimation of C may also be caused by preferential amplification of methylated alleles, though it occurs more rarely than the PCR bias favoring the unmethylated ones [44, 45]. Furthermore, it has been reported that tailed primers could introduce amplification bias depending on the template [46]. However, we can exclude these biases since the amplification of plasmid DNA standards harboring a G > A variant produced the expected G/A ratio. Nevertheless, we cannot rule out that the chosen tails, which work well with the two genes we analyzed, unevenly perform with other genes.

A number of studies have proposed alternative solutions for analyzing the Sanger-sequencing data, but their algorithms are often overwhelming [14, 44]. Our approach can be easily used and, importantly, it yields an estimate of methylation at each CpG site in agreement with data obtained with the conventional but cumbersome cloning-based method. Moreover, locus methylation as assessed by NBSP well reflected the miRNA expression in FFPE breast cancer samples. Importantly, NBSP allowed an accurate detection of methylation rate close to 10 %, a level below which methylation has negligible effects on miR-200c/miR-141 expression [47]. Finally, NBSP can be applied to other genes, such here shown for E-cadherin.

## Conclusions

We have presented here a reliable and cost-effective method to detect the methylation level of several CpGs. Our approach well performed in the analysis of the miR-200c/miR-141 locus and of the E-cad promoter, genes downregulated by methylation in a number of carcinoma. Besides, NBSP also works with FFPE tissues and thus may provide a viable and affordable tool to detect DNA methylation both for research and for diagnostic purposes.

## Additional files

Additional file 1: **Primers used in the methylation analysis.** (PDF 101 kb)
Additional file 2: **Amplification of miR-200/miR-141 locus performed with three couples of 5’-end tailed primers.** (PDF 96 kb)
Additional file 3: **Validation of the plasmid DNA standards.** (PDF 277 kb)
Additional file 4: **Amplification of miR-200c/miR-141 locus and methylation analysis.** (PDF 3603 kb)
Additional file 5: **miR-200c/miR-141 locus methylation of MDA-MB-231 breast cancer cell line determined by BSP and NBSP performed with forward and reverse primers.** (PDF 455 kb)
Additional file 6: **Inverse association between miR-200c/miR-141 locus methylation and miR-200c expression.** (PDF 1483 kb)

## Abbreviations

BSP: Direct sequencing of PCR from bisulfite-treated DNA
CGI: CpG island
E-cad: E-cadherin
EMT: Epithelial to mesenchymal transition
FFPE: Formalin-Fixed Paraffin-Embedded (tissue)
MBD-seq: Methylated DNA Binding Domain sequencing
MethylCap-seq: Methylated-DNA capture sequencing
NBSP: Normalized BSP
NF: Normalization factor
RRBS: Reduced representation bisulfite sequencing
WGBS: Whole-genome bisulfite sequencing
