# Supplementary material for: An improved sequencing-based strategy to estimate locus-specific DNA methylation
Source: BMC Cancer. 2015 Sep 21;15:639. doi: 10.1186/s12885-015-1646-6 (PMC4578270; doi:10.1186/s12885-015-1646-6)
Supplement: Additional file 1: — Primers used in the methylation analysis. (PDF 101 kb) [file 12885_2015_1646_MOESM1_ESM.pdf]

Additional file 1. Primers used in the methylation analysis.

**Untailed primer sequences**		
**Oligo name**	**Target locus**	**Sequence (5’->3’)**
200c-BSP-F *	miR-200c/miR-141 locus	TAGGTAAAGGTTATTAGGGGAGAGG
200c-BSP-R §	miR-200c/miR-141 locus	AACACTTCCTAATAAACCCTACTA
E-cad-BSP-F *	E-Cad promoter	GGAATTGTAAAGTATTTGTGAGTTTG
E-cad-BSP-R §	E-Cad promoter	AAATACCTACAACAACAACAACAAC
**5’-end tailed primer sequences for BSP and NBSP**		
**Oligo name**	**Target locus**	**Sequence (5’->3’)**
Tail1-200c-BSP-F *	miR-200c/miR-141 locus	CAGGAAACAGCTATGACCATAGGTAAAGGTTATTAGGGGAGAGG
**Tail2**-200c-BSP-R§	miR-200c/miR-141 locus	**TGACTGGTACGTACCAAC**AACACTTCCTAATAAACCCTACTA
Tail3-200c-BSP-F *	miR-200c/miR-141 locus	*TGCATGCAACGTCAGTGT*TAGGTAAAGGTTATTAGGGGAGAGG
Tail4-200c-BSP-R§	miR-200c/miR-141 locus	*CAACGTGTTGACGTGTCA*AACACTTCCTAATAAACCCTACTA
Tail5-200c-BSP-F *	miR-200c/miR-141 locus	*TGTGGTCACACAACACGT*TAGGTAAAGGTTATTAGGGGAGAGG
Tail6-200c-BSP-R§	miR-200c/miR-141 locus	*ACACGTACTGTGACGTCA*AACACTTCCTAATAAACCCTACTA
Tail1-E-cad-BSP-F*	E-Cad promoter	CAGGAAACAGCTATGACCAGGAATTGTAAAGTATTTGTGAGTTTG
**Tail2**-E-cad-BSP-R§	E-Cad promoter	**TGACTGGTACGTACCAAC**AAATACCTACAACAACAACAACAAC

The underlined characters represent the 5’-Tail1 (M13 reverse) added to the forward primers while the bold ones indicate the 5’-Tail2 (BC) added to the reverse primers; the italic characters represent the Tail 3, 4, 5 or 6; *F = forward primer, §R= reverse primer.
