# Supplementary material for: An improved sequencing-based strategy to estimate locus-specific DNA methylation
Source: BMC Cancer. 2015 Sep 21;15:639. doi: 10.1186/s12885-015-1646-6 (PMC4578270; doi:10.1186/s12885-015-1646-6)
Supplement: Additional file 2: — Amplification of miR-200/miR-141 locus performed with three couples of 5’-end tailed primers. (PDF 96 kb) [file 12885_2015_1646_MOESM2_ESM.pdf]

Additional file 2

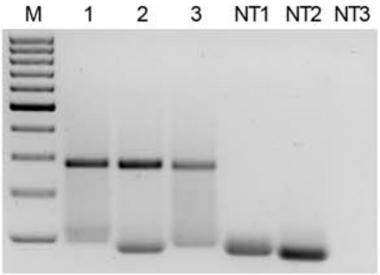

Amplification of miR-200c/miR-141 locus performed with three couples of 5'-end tailed primers.
5'-end tailed primers were obtained by adding Tail1, 3 and 5 at the 5'-end of the 200c-BSP-F and Tail2, 4 and 6 to the 200c-BSP-R. Amplification of MDA-MB-157 bisulfite treated DNA were performed with primers Tail1-200c-BSP-F/Tail2-200c-BSP-R (Lane 1), Tail3-200c-BSP-F/Tail4-200c-BSP-R (Lane 2) and Tail5-200c-BSP-F/Tail6-200c-BSP-R (Lane 3). Lane M, 100bp size marker. NT1, NT2, NT3, No Template controls relative to Lane 1, 2, 3, respectively.
