# Supplementary material for: An improved sequencing-based strategy to estimate locus-specific DNA methylation
Source: BMC Cancer. 2015 Sep 21;15:639. doi: 10.1186/s12885-015-1646-6 (PMC4578270; doi:10.1186/s12885-015-1646-6)
Supplement: Additional file 3: — Validation of the plasmid DNA standards. (PDF 277 kb) [file 12885_2015_1646_MOESM3_ESM.pdf]

Additional file 3

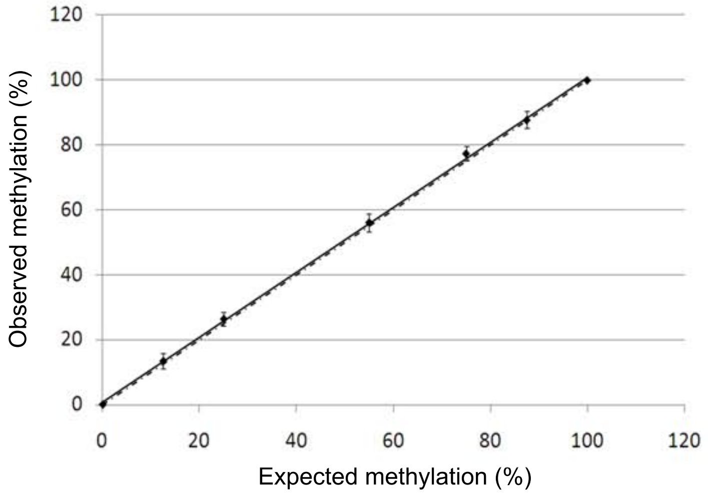

Validation of the plasmid DNA standards.

Plasmid DNA standards were directly sequenced and the observed methylation levels (average of the 14 CpGs) are plotted against the expected values. The solid line represents the linear regression and the dotted line the expected results.
