# Supplementary material for: An improved sequencing-based strategy to estimate locus-specific DNA methylation
Source: BMC Cancer. 2015 Sep 21;15:639. doi: 10.1186/s12885-015-1646-6 (PMC4578270; doi:10.1186/s12885-015-1646-6)
Supplement: Additional file 4: — Amplification of miR-200c/miR-141 locus and methylation analysis. (PDF 3603 kb) [file 12885_2015_1646_MOESM4_ESM.pdf]

Additional file 4

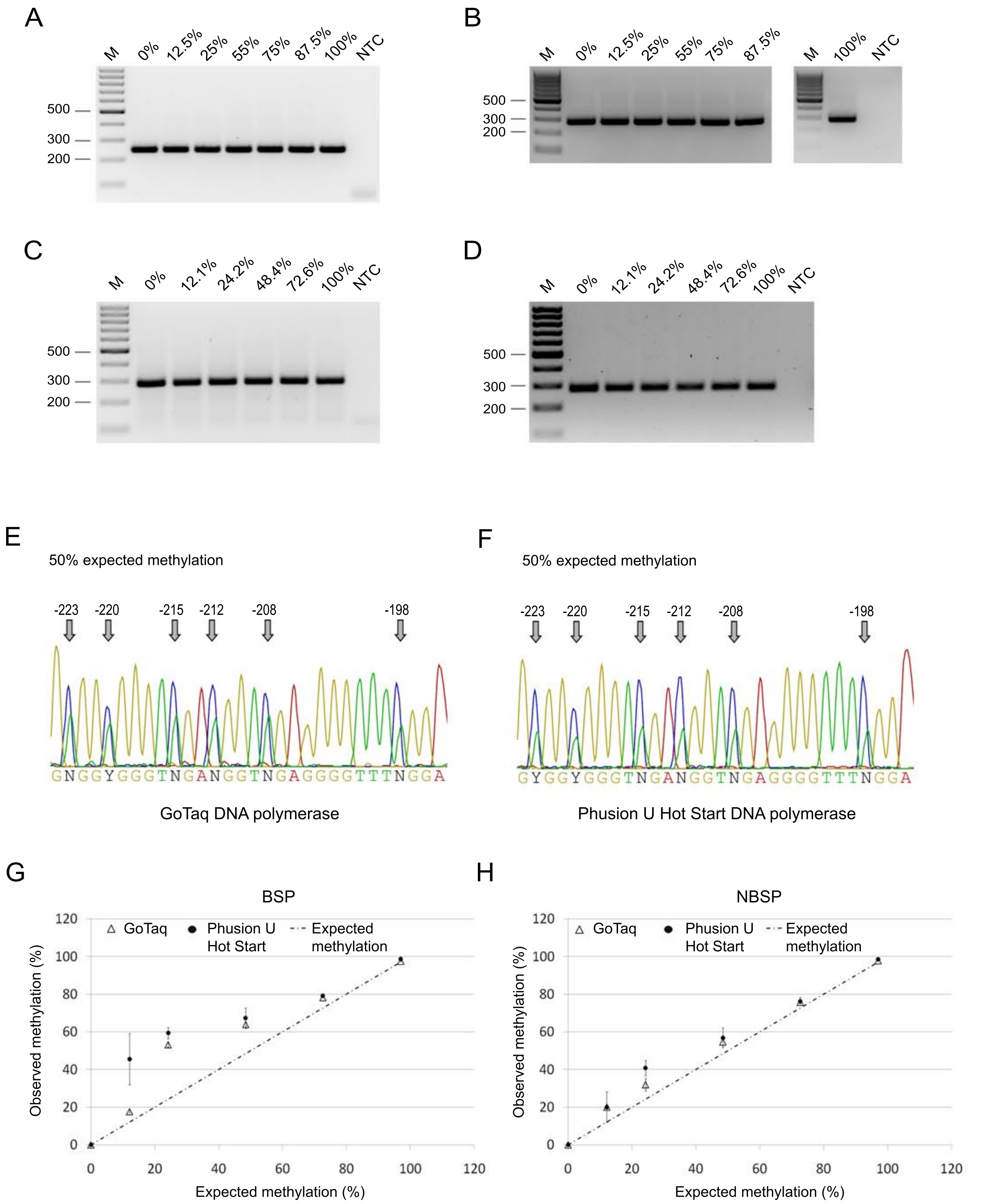

Amplification of miR-200c/miR-141 locus and methylation analysis.
Plasmid DNA standards were amplified with 200c-BSP-F/200c-BSP-R primers (**A**) or with Tail1-200c-BSP-F/Tail2-200c-BSP-R primers (**B**). Lane M, 100bp size marker. NTC, no template control. Genomic DNA standards were amplified with Tail1-200c-BSP-F/Tail2-200c-BSP-R primers by using GoTaq (**C**) or Phusion U Hot Start (**D**) DNA polymerases. Lane M, 100bp size marker. NTC, no template control. Representative sequencing chromatograms of a genomic DNA standard characterized by 50% CGI methylation and PCR performed with Tail1-200c-BSP-F/Tail2-200c-BSP-R primers by using GoTaq (**E**) or Phusion U Hot Start (**F**) DNA polymerases (Gray arrows indicate 6 out of 14 CpG analyzed). (**G-H**) The observed methylation levels (mean of the 14 CpG) of genomic DNA standards amplified with GoTaq (empty triangle) or Phusion U Hot Start (filled circle) DNA polymerases and assessed by using BSP (**G**) or NBSP (**H**) approaches are plotted against expected values. The dotted lines represent the expected methylation and bars indicate standard deviation**.**
