# Supplementary material for: An improved sequencing-based strategy to estimate locus-specific DNA methylation
Source: BMC Cancer. 2015 Sep 21;15:639. doi: 10.1186/s12885-015-1646-6 (PMC4578270; doi:10.1186/s12885-015-1646-6)
Supplement: Additional file 5: — miR-200c/miR-141 locus methylation of MDA-MB-231 breast cancer cell line determined by BSP and NBSP performed with forward and reverse primers. (PDF 455 kb) [file 12885_2015_1646_MOESM5_ESM.pdf]

# Additional file 5

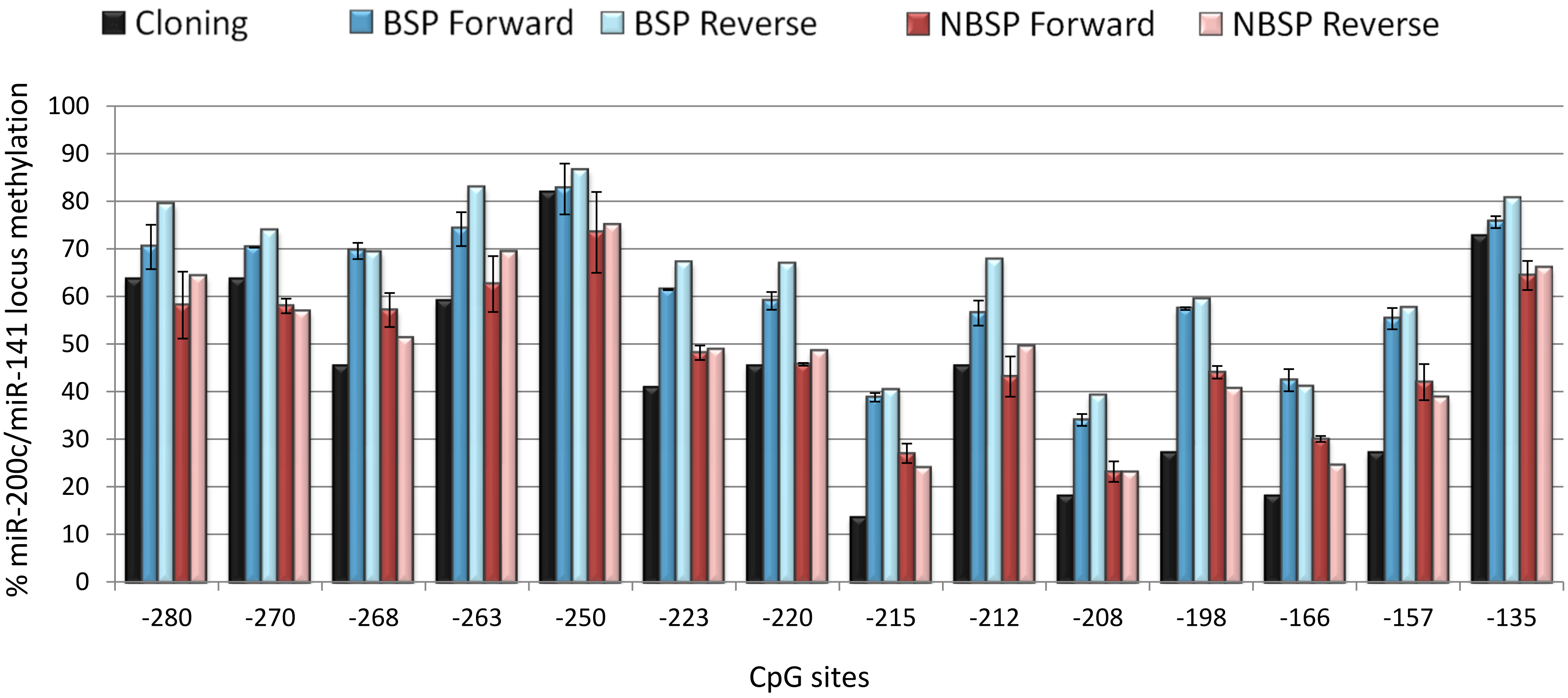


**miR-200c/miR-141 locus methylation of MDA-MB-231 breast cancer cell line determined by BSP and NBSP performed with forward and reverse primers.** In BSP Forward and NBSP Forward the oligo used for sequencing was Tail1; in BSP Reverse and NBSP Reverse the oligo used for sequencing was Tail2 (5'-TGACTGGTACGTACCAAC-3'). The methylation percentages of each CpG obtained from the cloning-based method (black columns), BSP Forward (blue columns), BSP Reverse (light blue columns), NBSP Forward (red columns), NBSP Reverse (pink columns) are reported.
