# Supplementary material for: An improved sequencing-based strategy to estimate locus-specific DNA methylation
Source: BMC Cancer. 2015 Sep 21;15:639. doi: 10.1186/s12885-015-1646-6 (PMC4578270; doi:10.1186/s12885-015-1646-6)
Supplement: Additional file 6: — Inverse association between miR-200c/miR-141 locus methylation and miR-200c expression. (PDF 1483 kb) [file 12885_2015_1646_MOESM6_ESM.pdf]

Additional file 6

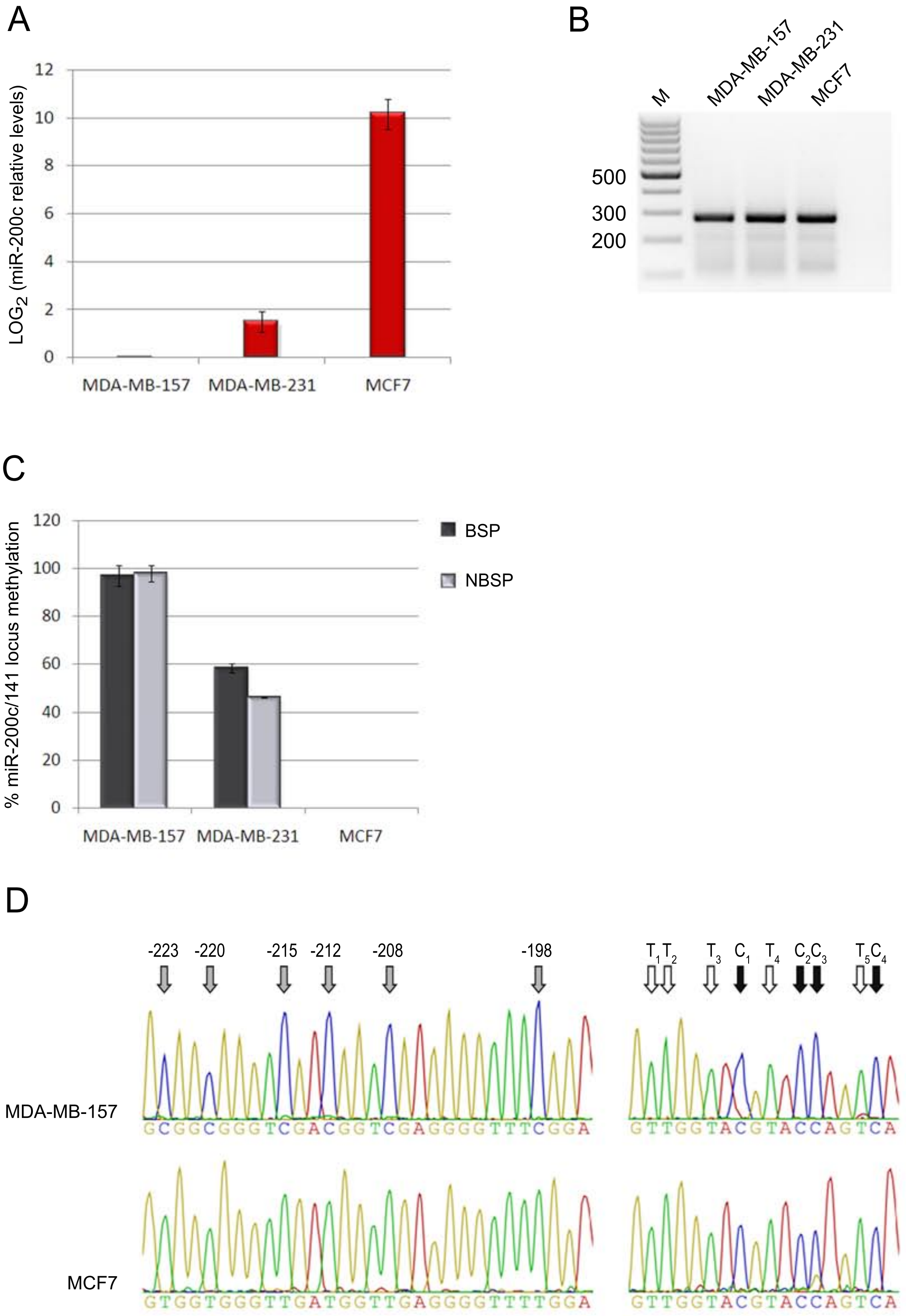

Inverse association between miR-200c/miR-141 locus methylation and miR-200c expression.

(**A**) miR-200c levels of MDA-MB-157, MDA-MB-231 and MCF7 cell lines were determined by qRT-PCR and reported as the LOG2 of the miR-200c relative expression levels. (**B**) miR-200c/miR-141 locus PCR of bisulfite treated DNA obtained from the MDA-MB-157, MDA-MB-231 and MCF7 cell lines. Lane M, 100 bp size marker; NTC, no template control. (**C**) miR-200c/miR-141 locus methylation levels of MDA-MB-157, MDA-MB-231 and MCF7 cell lines measured by BSP (black columns) and NBSP (gray columns). Data reported are the averages of methylation rate of the 14 CpG. (**D**) Representative sequencing chromatograms of 6 CpG (highlighted by gray arrows; left panels) and of the reverse sequence of Tail2 of miR-200c/miR-141 amplicon (right panels; with C and T used to calculate the NF indicated by black and white arrows, respectively) for MDA-MB-157 (upper panels) and MCF7 (lower panels).
